# Complete Heart Block in a Diabetic Patient with a Preexisting LBBB and Normal Coronaries, Paradoxically Responding to Atropine

**DOI:** 10.1155/2018/2459691

**Published:** 2018-12-30

**Authors:** Nikolaos S. Ioakeimidis, Dimitrios Valasiadis, Lykourgos Nanis, Pantelis Kligkatsis, Stefanos Papastefanou

**Affiliations:** ^1^Department of Cardiology, General Hospital of Florina “Eleni Th. Dimitriou”, Egnatias 9, Florina 53100, Greece; ^2^Department of Cardiology, General Hospital of Thessaloniki “Agios Pavlos”, Ethnikis Antistaseos Avenue 161, Thessaloniki 55134, Greece

## Abstract

We present a case of a complete atrioventricular block (AV block) with different aberrancy patterns during sinus rhythm and escape rhythm. A 66-year-old woman visited our emergency department complaining of sudden onset dizziness and fatigue over the past thirty minutes. Her medical history was remarkable for arterial hypertension, type 2 diabetes mellitus, and hypothyroidism. The patient had a known Left Bundle Branch Block (LBBB) on past ECGs. Upon palpation of peripheral pulse, a measurement of 32 beats per minute was obtained. No other sign of hemodynamic instability was present. A 12-Lead ECG revealed a complete heart block with sparse QRS complexes with a Right Bundle Branch Block (RBBB) morphology. Before the insertion of a temporary transvenous pacemaker, atropine was administered intravenously. Shortly after the administration, the patient's heart rhythm was restored to sinus rhythm (SR) with LBBB. The patient remained hemodynamically stable and in sinus rhythm at the cardiac ICU and was scheduled for implantation of a permanent pacemaker at a specialized tertiary center. Before successful implantation, a coronary angiography revealed normal coronary anatomy with no atherosclerotic lesions.

## 1. Introduction

Complete heart block or 3rd degree atrioventricular block is a clinical entity described in case series from the early years of electrocardiography [1]. Its prevalence is quite low at approximately 0.04% in the general population [2]. However, it is found to be increased in patients with type 2 diabetes mellitus [3].

## 2. Case Presentation

**Figure 1:**
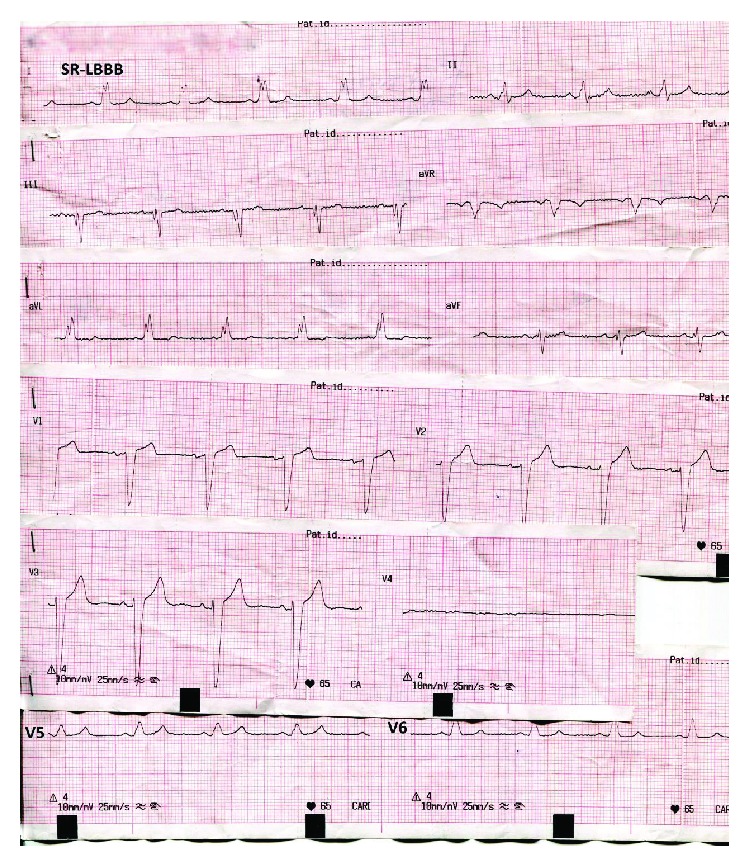
The patient's ECG six months before her arrival at our emergency department with a complete heart block. Sinus rhythm with LBBB.

**Figure 2:**
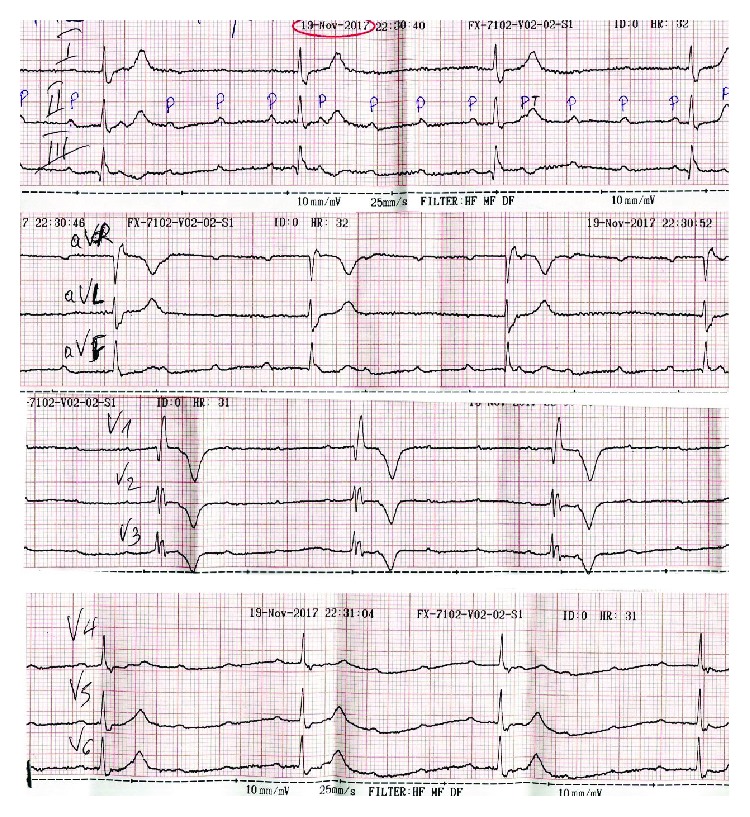
The patient's ECG at presentation. Complete heart block with an atrial rate of approximately 120 bpm and a ventricular rate of 32 bpm.

**Figure 3:**
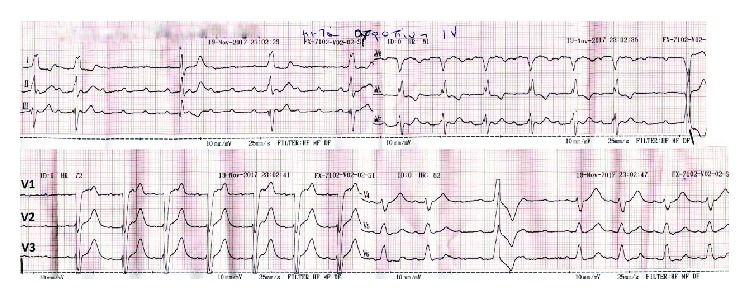
The patient's 12-Lead ECG after intravenous atropine administration. Sinus rhythm with LBBB.

**Figure 4:**
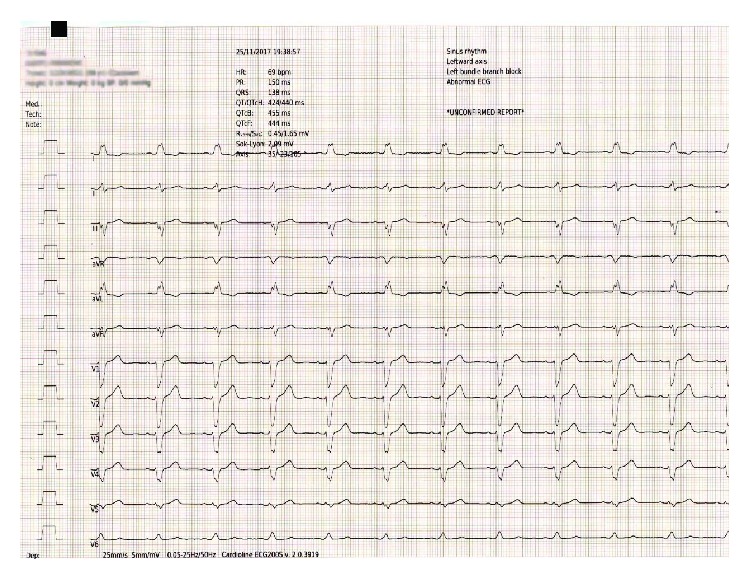
The patient's discharge ECG before her transfer to a specialized center for pacemaker implantation. Sinus rhythm with LBBB.

**Figure 5:**
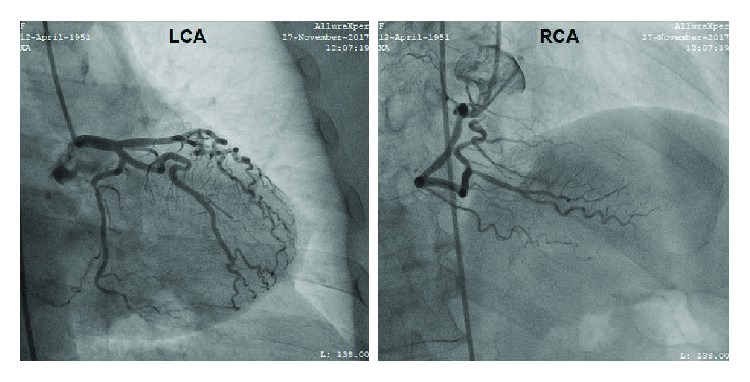
Coronary angiography which revealed no atherosclerotic lesions.

A 66-year-old woman presented at the emergency department of our hospital complaining of sudden onset dizziness and fatigue over the past thirty minutes. Before her arrival, she was at home relaxing and not engaged in any physical activity. Her past medical history was significant for arterial hypertension, diabetes mellitus, and hypothyroidism. Her medications were tab. vildagliptin/metformin (50/1000) (mg) BID, tab. amlodipine/valsartan (5/160) (mg) once daily, and tab. levothyroxine 75 mcg once daily. She had a known and asymptomatic Left Bundle Branch Block (LBBB) and a normal echocardiogram on previous regular visits at her cardiologist (Figure 1). Upon palpation of peripheral pulse, a measurement of 32 beats per minute (bpm) was obtained. Her blood pressure was 115/60 millimeters of mercury and her oxygen saturation 96% on room air. A 12-Lead ECG was recorded and revealed a complete heart block (CHB) with sparse QRS complexes with a Right Bundle Branch Block (RBBB) morphology (Figure 2). Before the insertion of a temporary transvenous pacemaker, atropine 2 mg was administered intravenously as a bolus infusion. Shortly after, sinus acceleration was observed and conversion of the complete AV block into 2nd degree AV block with 2 : 1 conduction (note that the blocked P waves are more visible in Lead V1) (Figure 3). Eventually, her heart rhythm was restored to SR with LBBB, at approximately 72 bpm (Figure 3). Laboratory studies revealed a normal complete blood count, normal electrolytes, cardiac enzymes, and Thyroid Stimulating Hormone (TSH). The patient was immediately transferred to the cardiac intensive care unit, hemodynamically stable and under continuous ECG monitoring. Her stay at our clinic remained uneventful until her transfer to a specialized tertiary center for a permanent pacemaker implantation (Figure 4). Apart from the implantation, a coronary angiography was performed which revealed normal coronary arteries without atherosclerotic lesions (Figure 5).

## 3. Discussion

Regarding the substrate of our patient's CHB, it is important to mention that increased prevalence of high-degree atrioventricular block in diabetic patients is reported by numerous studies [3–5]. However, randomized prospective studies on the matter are lacking. The pathophysiological mechanisms behind the association of DM with CHB remain largely unclear, with possible explanations including diabetic microangiopathy or progression of bundle branch blocks to a CHB [6]. The clinician, regardless of specialty, should be well aware of the implication of DM in cardiac conduction system abnormalities.

The effect of atropine is known to improve AV block at the nodal level and not at the level of the His-Purkinje system [7]. However, in the presented case, the preexistent LBBB during SR and the escape rhythm at a very slow rate (about 30 bpm) with a wide QRS pattern are all suggestive of an infranodal level of this AV block. The mechanism underlying the response to atropine is unknown in this particular case and might be due to vagolytic effect in the context of a “diseased” His-Purkinje system. In the presence of a 2 : 1 AV block, as our patient exhibited after atropine administration, it can be perplexing to differentiate nodal from infranodal block by ECG interpretation alone. During the brief 2 : 1 AV block, we notice that the PR interval is approximately 140 ms, smaller than 160 ms, suggesting that the block is localized in the bundle of His or bundle branches [8]. The most probable origin of the escape rhythm is the left bundle branch distally to the site of block and thus resulting in a typical RBBB morphology. An invasive electrophysiological study would have been proven extremely useful in clarifying the exact substrate of the patient's complete heart block.
